# Moving backwards, moving forward: the experiences of older Filipino migrants adjusting to life in New Zealand

**DOI:** 10.1080/17482631.2017.1347011

**Published:** 2017-07-13

**Authors:** Jed Montayre, Stephen Neville, Eleanor Holroyd

**Affiliations:** ^a^ Nursing Department, School of Clinical Sciences, Auckland University of Technology, Auckland, New Zealand; ^b^ Melbourne School of Population and Global Health, University of Melbourne, Victoria, Australia

**Keywords:** Older migrants, Filipinos, adjustment, life experiences, New Zealand, qualitative

## Abstract

**Purpose**: To explore the experiences of older Filipino migrants adjusting to living permanently in New Zealand.

**Method**: The qualitative descriptive approach taken in this study involved 17 individual face-to-face interviews of older Filipino migrants in New Zealand.

**Results**: Three main themes emerged from the data. The first theme was “moving backwards and moving forward”, which described how these older Filipino migrants adjusted to challenges they experienced with migration. The second theme was “engaging with health services” and presented challenges relating to the New Zealand healthcare system, including a lack of knowledge of the nature of health services, language barriers, and differences in cultural views. The third theme, “new-found home”, highlighted establishing a Filipino identity in New Zealand and adjusting to the challenges of relocation.

**Conclusion**: Adjustment to life in New Zealand for these older Filipino migrants meant starting over again by building new values through learning the basics and then moving forward from there.

## Introduction

Historically, global migration trends have shown a significantly lower proportion of older people migrating compared to younger age cohorts (United Nations [UN], 2016). However, more recently, the global migrant profile has no longer been dominated by the young working-age population and it now includes a large percentage of older adults (UN, 2016; Warnes & Williams, 2006). The increasing number of older migrants is illustrated by the higher percentage of migrants who are 60 years old and over in proportion to the total population of the same age group among major migrant destination countries (UN, 2016). This rise has been fuelled by an increased life expectancy and legal migrant pathways for reunification (Zaiceva, 2014).

In recent years, a considerable number of parents of New Zealand residents of different ethnicities have been granted residency status (Immigration New Zealand Statistics, 2013). In New Zealand, studies focusing on South Korean, Chinese, South African, and Indian migrants have been undertaken (Alpass et al., 2007; DeSouza, 2006; Park & Kim, 2013), revealing migration issues and processes affecting migrants’ psychological well-being and their experiences of growing old in New Zealand. Furthermore, there has been an increase in empirical work documenting the complex health and well-being needs of older migrants, who are culturally and linguistically diverse (Al Abed, Davidson, & Hickman, 2014; Radermacher, Feldman, & Browning, 2009). Ageing in general is accompanied by inevitable and complex co-morbidities, including physiological and psychosocial health-related issues. These health complexities are experienced by older migrants more acutely than by younger migrants (Khoo, 2012). Nikolova and Graham (2015) noted that migration to developed economies, while increasing subjective well-being for the younger population groups, often meant that older migrants experience an interrupted social connectedness, referred to as a “broken social convoy” (Park et al., 2015).

Filipinos are recognized as one of the most outwardly migrating populations in the world. As early as 1907, Filipinos were leaving the Philippines to live and work overseas. Every year, an increasing number of Filipino workers migrate to seek better opportunities in more resource-rich countries (Philippine Overseas Employment Administration, 2016). The sociodemographic profile of Filipino migrants in recent years has documented large numbers of young professionals from various industries such as healthcare and engineering, as well as unskilled workers in the form of domestic helpers, transport operators, and labourers (Ball, 2004; Philippine Overseas Employment Administration, 2016). Since the 1970s, almost 10 million Filipinos have relocated overseas for such reasons as work and cross-cultural marriages, and through family reunification programmes, where parents are granted citizenship by the adopting country allowing sponsorship for children or vice versa (Philippine Overseas Employment Administration, 2016). It has also been noted that increasing numbers of Filipino migrants aged 60 years and over are living permanently in major migrant-hosting countries (UN, 2016).

New Zealand is arguably one of the most culturally diverse countries in the world and constitutes a recent new destination for Filipino migration (Statistics New Zealand, 2015). Compared to other destination countries such as the USA, Filipino migration to New Zealand has steadily increased in the past 30 years (UN, 2016). The 2015 New Zealand census revealed the varied characteristics of migrants, with an increasing number of older migrants, mostly from Asia, including Filipinos (Statistics New Zealand, 2015). Filipinos have been engaged in “roving or circular” migration patterns, involving migrating to one country and then to another, mainly for employment and economic reasons (Commission on Filipinos Overseas, 2013; International Organization for Migration (IOM), 2013b). However, what makes New Zealand remarkable among the destination countries for Filipinos is that it belongs to the top 10 countries where Filipinos prefer to live permanently (Commission on Filipinos Overseas, 2013).

However, there have been limited studies on the adjustment processes of older Filipino migrants, despite this group comprising the third largest of the Asian ethnic groups in New Zealand. The lack of research in this area resonates with the recommendation of the World Migration Report 2013 on Migrant Well-being and Development, highlighting the importance of listening to migrant stories and their perceptions and experiences of migrating to a new country (IOM, 2013a). Migrants’ narratives hold the key to understanding their well-being and specific cultural needs, which have critical implications for policy-making in their host countries (IOM, 2013b). One relevant example concerns healthcare policies for migrant families and ethnic groups, which undergo comprehensive background work and a careful analytical decision-making process at the policy level (Mladovsky, 2009). The increasing global population of older migrants and the multifaceted implications identified in the growing body of literature on the topic from different disciplines (Ciobanu, Fokkema, & Nedelcu, 2017) suggest a need for further exploration through empirical research.

The main objective of this study is to explore the experiences of older Filipino migrants adjusting to life in New Zealand.

## Method

### Design

This research utilized a qualitative descriptive approach to understand the challenges and experiences faced by older Filipino migrants adjusting to life in New Zealand. According to Sandelowski (2000), a descriptive qualitative approach is the “method of choice when straight descriptions of the phenomena are desired” (p. 339). The theoretical and epistemological position of this methodology draws from the “general tenets of naturalistic inquiry” (Sandelowski, 2000, p. 337). Naturalistic inquiry explores a phenomenon exclusive to its natural state, which enables researchers to gain an understanding of participants’ perceptions about real-life situations (Guba & Lincoln, 1982), and this type of inquiry aligns with qualitative description, where findings are presented close to the participants’ actual words, referred to as “data-near” (Sandelowski, 2010). A naturalistic philosophical orientation allowed the researchers in this study to undertake an analysis that sought “both descriptive validity, an accurate accounting of events that most people observing the same event would agree is accurate, and interpretive validity, or an accurate accounting of the meanings participants attributed to those events that those participants would agree is accurate” (Sandelowski, 2000, p. 336). In addition, there is a paucity of research on older Filipinos’ ageing and migration experiences, which positions qualitative descriptive methodology perfectly in collecting rich descriptions of these phenomena.

### Sampling

Participants were selected using a purposive sampling design. Purposive selection deploys inclusion and exclusion criteria to recruit participants who have the requisite sociodemographic background to address the aim of the study (LoBiondo-Wood & Haber, 2014). Inclusion criteria for participation in the present study included: (1) being a Filipino migrant aged 60 years old or over, (2) having migrated to New Zealand aged 60 years or over, (3) having a permanent resident visa condition, and (4) having lived in New Zealand for at least 2 years.

### Ethics

Ethics approval was granted by the Auckland University of Technology ethics committee (reference number 016-42). Written consent was obtained from each participant before undertaking the face-to-face interviews. The specific names reported with the corresponding excerpts in the current research were pseudonyms assigned by the researchers to maintain anonymity and to conceal the participants’ real names and identities.

### Recruitment and data collection

Recruitment took place in a small provincial city in Southland, New Zealand. Potential participants were approached via a Filipino community centre where older adults attended church and social gatherings. Twenty participants who met the inclusion criteria were initially approached; however, three declined because they were busy with family commitments, leaving 17 participants who were interviewed.

All interviews were conducted in Filipino (the national language of the Philippines) by the primary author, who is a Filipino and fully fluent in both English and Filipino. The interviews were digitally recorded and lasted for 30–45 min. The semi-structured interview guide included questions such as “How did you find relocating to New Zealand at your age?”, “Are there any challenges that you have experienced upon migrating here?”, and “How did you manage and adjust to these?” All raw interview data were concurrently transcribed verbatim and translated into English by an independent bilingual translator. All data were read, reread, and then coded independently by two members of the research team.

### Data analysis

An inductive and data-driven thematic analytic process was undertaken to identify repeated patterns of meaning (Braun & Clarke, 2006). This method of analysis of qualitative data is an “accessible and theoretically flexible approach” (Braun & Clarke, 2006, p. 77), which provides a clear step-by-step process for the current research underpinned by a naturalist epistemology.

Thematic analysis as utilized in this study identified patterns “within and across” interview data in relation to the participants’ experiences, views, and perspectives (Clarke & Braun, 2017, p. 297). The analytical process begins with the identification of patterns of language and words used by older Filipino migrants and the meaning attached to these, then moves towards the identification of implied constructs that were articulated by the participants. The technical process of analysis involved transcription of data, reading, repeatedly reading the transcripts, capturing the interesting features of the data (coding), and identifying patterns or themes following the Braun and Clarke (2006) analysis framework. The authors then reviewed the provisional themes, and discussed further refinement of the coding and analysis until they identified and agreed upon the salient patterns repeated across and within transcripts (Braun & Clarke, 2006).

### Rigour

The interviews were conducted in the Filipino language, and care was taken to ensure accurate translation to English (Temple & Young, 2004). Two transcripts were back-translated by an independent bilingual Filipino researcher from another university to ensure the accuracy of the transcripts and in order not to lose the meaning of the original language (Brislin, 1975). The original transcripts and back-translated versions were compared for accuracy of translation, agreeing on Filipino terminologies that do not have direct English-language equivalents, and ensuring that modifications were made accordingly. Peer-checking by co-researchers was also undertaken to enable the accurate identification of themes.

## Results

### Participant demographics

Seventeen older Filipino migrants were interviewed, comprising 10 females and seven males. Fourteen of the participants were aged between 60 and 65 years old and three were over the age of 65 years. At the time of the interview, seven participants had been living in New Zealand for 2 years, seven had been in the country for 3–5 years, and three participants had been living there for more than 6 years.

### Themes

Three main themes emerged from the data. The first theme, “moving backwards and moving forward”, incorporates the adjustment process of older Filipino migrants to challenges they have experienced upon migrating to New Zealand. The second theme,“engaging with health services’, presents some of the challenges experienced when engaging with the New Zealand health system. The third theme, “new-found home”, highlights establishing a Filipino identity in New Zealand, and portrays older Filipino migrants adjusting to the challenges brought about through the relocation process.

#### Moving backwards and moving forward

Older Filipino migrants recognized that migrating to New Zealand had a significant impact on their personal lives. Initially, participants described their lives as “moving backwards”, which relates to the new environment requiring them to re-engage in activities and relearn skills they had already mastered in their home country but in a culturally different context. On the other hand, they acknowledged that while initially migration had disrupted their lives, it had also served as a catalyst to plan for the future, which was referred to in the interviews as “moving forward”:

We felt like we went back to the very beginning, starting all over again with our life. We are very pleased to be with our family here but it was never easy to feel new and unfamiliar. (Josefina)

In the first few months here, I woke up every morning wondering what new things I would learn or see today. We went to the supermarket, I always felt that even though I know what to expect when shopping, I just felt that they may do something different here. (Maria)

An older Filipino male migrant said:

I never used a map to get to places, in fact I am not very good at using maps. However, I had to learn how to use one and get directions as I am new to the place, although my daughter always accompanies me when I go out. I insist that I need to be accustomed with how things work here. I need to know the basics, particularly with transport. (Pedro)

Moving “backwards” was also expressed in the context of personal regression followed by acceptance, as seen in the following excerpt:

We came here for a fresh start, as we look to the future of getting old here. We recognize we must start again with no time for regrets, regardless of what we used to enjoy back in the Philippines. We’re like a tirador [slingshot], you have to be pulled back to move forward. (Lolita)

The participants appreciated the positive experiences that migration had brought to their lives, which contributed to their “moving forward”, as identified in the following excerpt:

If I stayed in the Philippines, I might not be able to see the world like what I am doing now. I have travelled most places in the world. It was always my dream to travel. I would have waited forever to do that with my Philippine passport. (Maria)

Achieving and learning new skills meant moving ahead and realizing goals:

I feel like I have developed better life skills living in this country. Skills that I may not have developed if we remained in the Philippines. I always wanted to drive despite my advancing age. I have learned how to drive here. Back home, my husband drove me places. Here all of my daughters are working so for me to get around, I had to learn to drive and I learned fast. (Sylvia)

“Moving forward” was frequently linked to having achieved a way of living that could only be dreamed about when living in the Philippines:

We worked so hard back home. My son moved to New Zealand and lives in this beautiful house with the beach view. My son and his family asked us to live with them in this wonderful house by the sea … we could only dream of living in a house like this in the Philippines. (Pedro)

#### Engaging with health services

Accessing health services is integral to supporting health and well-being for older people. Mechanisms and processes for engaging with health services vary widely between countries and ethnicities. In New Zealand, general practitioners are central to the functioning of health services. For some older Filipino migrants, their initial engagement with the New Zealand health system was confusing:

I hurt my ankle when I slipped on snowy ground and I couldn’t walk because of the pain. We went to Emergency and we were very new and my English was not very good. I could hardly understand what the nurse at reception was saying. We waited and waited in the waiting lounge with my painful foot, only to be told that we needed to see a general practitioner, which I now realize is another doctor. We don’t call our doctors general practitioners at home. I told my husband, why do they send us to another doctor? I presumed they have doctors in the emergency room. It was only after the incident, when a friend of mine who’s been here longer than us, explained about enrolling in a general practice. (Sandra)

Many participants in this study felt that their cultural practices were not taken into account by hospital staff. One woman provided the example of not being able to stay with her daughter, who was having a baby:

I wanted to stay overnight in the hospital when my daughter gave birth. I was told I could not and the policy is you come and visit during visiting hours but don’t stay overnight. Back home, when someone is in the hospital especially immediate family, we are allowed to stay, here rules are different. (Hilda)

In the Philippines, it is accepted practice to stay with and be the main provider of care to a family member in hospital:

Back home we help our sick family members when they are hospitalized by staying in the hospital and help them by providing care. Here, if the room gets crowded with people, it’s a risk for infection and if you want to stay it is not allowed. (Celia)

Participants who had engaged with the New Zealand healthcare system, particularly those who had been hospitalized, spoke of feeling ignored because English was not their first language:

I was always waiting for a Filipino nurse to pass by my bed so I could ask her to help me with something as I wasn’t able to get what I needed from the other nurses. The nurses were told that I had limited English so they very rarely talked to me, instead they would always call the Filipino nurse, and when that Filipino nurse started talking to me in English and I responded back, the other nurses were surprised that I could understand English. (Lina)

#### New-found home

Overall, these older Filipino migrants considered New Zealand to be their “new-found home”. They described adjusting to their newly adopted home by learning to live the New Zealand way while remaining connected to their Filipino cultural identity.

Living the New Zealand way meant that they had successfully adjusted to life in New Zealand. However, one woman who had been in New Zealand for only 2 years had found life challenging at the start:

My son told me about the differences of New Zealand culture to the one I grew up with. Although I got to know now how to respond and what to expect, it was difficult when I actually experienced these differences myself. With my previous job back in the Philippines as a sales agent, I am connected with different types of people and communicated effectively, but here, I did not experience those same connections. I think when you are new to a place with a different culture you start from scratch regardless of what you have previously achieved in your life or in your previous job. (Nora)

Another pointed out how she adapted to living the New Zealand way by learning simple social gestures:

Over time, you get to learn how to live the Kiwi way. One example is when you attend a party or gathering even if it is a Filipino party. We are getting used to the culture of bringing a plate and sharing it with everybody. The host of the party does not need to cook or prepare everything the visitors eat, like what happens in the Philippines where visitors arrive empty-handed. (Celia)

The ability to adapt to the New Zealand way of life while still preserving one’s own cultural identity was noted in the following excerpt:

I still cook and eat rice, although when you attend parties hosted by Kiwis, you don’t expect they will have rice at those parties, you need to learn how things work here as this is where I live now. (Tomas)

Integral to making New Zealand home was remaining connected to their Filipino heritage, especially through buying food, as outlined in the following excerpts:

We shop in the Filipino store or Asian shops. In those shops I feel like I am in control as I know what to buy, I know the products and their quality. However, sometimes I get reminded it is very expensive. However, I miss Filipino food so I don’t care how much it costs. (Belen)

We went to a Filipino restaurant in Dunedin, I really enjoyed the food, and the atmosphere, and there were a lot of Filipinos talking in Tagalog. I felt like I was in the Philippines. I thought when I get homesick I just have to come here and eat and it feels like home. (Miguel)

## Discussion

This study explored the experiences of older Filipino migrants and the challenges they faced when adjusting to living permanently in New Zealand. Older Filipino migrants spoke about having to adjust to living in a new environment and relearning ways of doing things in another country. This adjustment was referred to in the present study as “moving backwards”. This is supported by Park and Kim (2013), who describe older migrants’ experience of disconnection from previous life routines back in their country of origin as part of the adjustment process. Similar migrant experiences have also been noted by Park et al. (2015), who refer to the “broken social convoy effect”, suggesting one of the many social vulnerabilities encountered by older migrants in their host societies, compounded by their age-related changes and life experiences (Ciobanu et al., 2017). Over time, the older Filipino migrants interviewed in the present study felt that they had adjusted to the challenges of living in a new country and had learnt to appreciate and enjoy living in New Zealand; this was referred to as “moving forward”. Their optimism about living in New Zealand suggests that this cohort of older Filipinos felt that they were effectively managing and adjusting to the challenges they had experienced, and were starting to enjoy the benefits of having migrated to a new country. Older Filipino migrants described overcoming challenges in a context of using a “tirador” (slingshot), which means moving backwards or starting over in order to move forwards. Other migrant groups in New Zealand have also experienced such a transition, from challenging times to having adapted to and feeling settled living in New Zealand (Park & Kim, 2013; Winbush & Selby, 2015).

This cohort of older Filipino migrants found engaging with the New Zealand health system challenging. Key challenges included a lack of knowledge of the nature of health services, language barriers, and differences in their own cultural views towards health and healthcare professionals. Older Filipino migrants’ health practices are known to be influenced by traditional beliefs and the use of herbal remedies, which affect their willingness to access health services (Cruz & Periyakoil, 2010; Dela Cruz & Galang, 2008). Life expectancy among older people residing in the Philippines is shorter than that of older Filipino migrants in major adopting countries (World Health Organization, 2016). Commonly, the healthcare system in the destination country is better than that of the home society; however, migrants still experience challenges and difficulties with communication and cultural differences (Ozolins & Hjelm, 2003). Language and cultural barriers between patients and healthcare professionals compound the complexities among migrants accessing healthcare services (Aelbrecht, Pype, Vos, & Deveugele, 2016).

The narrative accounts of these older Filipino migrants suggest a need for clear health and social service provision policies to enable positive migrant adjustment upon arrival in New Zealand. The lack of knowledge about health and social services is common among older migrants, leading to issues such as the underutilization of available services (Bolzman, Fibbi, & Vial, 2006). Healthcare agencies need an increased awareness of the changing demographics of New Zealand’s older health consumers, require responsive and contemporary educational knowledge on migrant health and social needs, and need to provide culturally targeted services. The experiences of older Filipinos in the healthcare system indicate the need for integrated and enhanced migrant health services specific to Filipinos. The need for culturally targeted services is supported by evidence from a study of the effects of ethnicity and culture on the well-being of older migrants (de Jong Gierveld, Van der Pas, & Keating, 2015).

The findings from the current study identified that most of these participants considered New Zealand their new home. The process of remaining connected to Filipino heritage and culture is integral to migrants adjusting to life in a host country (Berry, 1990). This cohort of older Filipino migrants chose to eat traditional foods, shop in Filipino and Asian stores, and socialize with other Filipino groups as ways of remaining connected to their cultural identity. These activities helped with adjusting to life in a new country. This finding of holding “onto home” has also been evident in studies among other migrant groups in New Zealand (Giorgio, 2015). Adapting to living in a host country was a time-consuming process, with migrants undergoing a transition period as part of the adjustment process. Filipino migrants adjust to living in Western society by a complex process of learning to speak and understand English, as well as assimilating their religious beliefs and practices into their host countries (Clyne & Kipp, 2003). This cohort of older Filipinos identified an ability to make successful positive and highly personalized adjustments through gradual assimilation and integration into New Zealand society.

### Limitations

This study was undertaken with and limited to a group of older Filipino migrants residing in one provincial city in southern New Zealand. Extending the geographical scope of the research to large regional and metropolitan towns in future studies could produce more transferable findings. A more balanced representation of male and female participants would also improve the heterogeneity of the study.

## Conclusion

This research is the first to focus on the migration experiences and, in particular, health system challenges of older Filipinos in the New Zealand context. Adjustment to life in New Zealand for older Filipino migrants meant starting over and building new values by learning the basics and then moving forward from there, while retaining ties through communities and Filipino traditions. Challenges with healthcare engagement were evident during the initial stages of adjustment and gradually progressed to a positive adjustment as these older Filipinos finally considered adapting to New Zealand as their new home.
